# Multi-Type Stochastic Resonances for Noise-Enhanced Mechanical, Optical, and Acoustic Sensing

**DOI:** 10.34133/research.0386

**Published:** 2024-05-30

**Authors:** Zhu Liu, Kai Qu, Ke Chen, Zhipeng Li

**Affiliations:** ^1^ School of Physics and Electronics, Hunan Normal University, Changsha 410081, China.; ^2^ Key Laboratory of Physics and Devices in Post-Moore Er, College of Hunan Province, Changsha 410081, China.; ^3^ School of Electronic Science and Engineering, Nanjing University, Nanjing 210023, China.; ^4^ Department of Electrical and Computer Engineering, National University of Singapore, Singapore 117583, Singapore.

## Abstract

Stochastic resonance (SR) typically manifests in nonlinear systems, wherein the detection of a weak signal is bolstered by the addition of noise. Since its first discovery in a study of ice ages on Earth, various types of SRs have been observed in biological and physical systems and have been implemented in sensors to benefit from noise. However, a universally designed sensor architecture capable of accommodating different types of SRs has not been proposed, and the widespread applications of SRs in daily environments have not yet been demonstrated. Here, we propose a sensor architecture to simultaneously realize multi-type SRs and demonstrate their wide applications in mechanical, optical, and acoustic sensing domains. In particular, we find the coexistence of excitable SR and bistable SR in a sensor architecture composed of wirelessly coupled inductor–capacitor resonators connected to a nonlinearly saturable amplifier. In both types of SRs, adding noise to the system leads to a characteristic noise-enhanced signal-to-noise ratio (SNR). We further validate our findings through mechanical, optical, and acoustic sensing experiments and obtain noise-enhanced SNR by 9 dB, 3 dB, and 7 dB, respectively, compared to the standard methods devoid of SR integration. Our findings provide a general strategy to design various types of SRs and pave the way for the development of a distinctive class of sensors leveraging environmental noise, with potential applications ranging from biomedical devices to ambient sensing.

## Introduction

Sensors play an important role in many technologies used in daily life, spanning from wearable health monitors to ambient sensors in the Internet of Things. Deploying sensors in daily environments, however, is challenging due to the ubiquitous presence of noise. Because noise usually interferes with signal detection, most approaches to noise mitigation, such as spectral filtering, artifact removal, and active cancellation [1], primarily focus on minimizing or eliminating noise. An alternative approach involves engineering the system’s response to a noisy input signal [2], a strategy found in various biological sensory systems where noise paradoxically enhances the capability of nonlinear systems to detect weak signals—an effect known as stochastic resonance (SR) [3,4].

SR operates on the principle of a system’s sensory threshold and is characterized by 2 primary types based on different sensory threshold models: excitable SR and bistable SR [5–7]. These types of SRs have been implemented across a wide range of physical systems, including optomechanical resonators [8–10], photonic semiconductors [11], and bistable circuits [12]. Biomedical applications have also harnessed SRs to enhance balance control [13,14], auditory perception [15], and tactile sensation [16,17]. Despite these advancements, SRs remain confined to respective physical systems, and its integration into a broader class of sensors operating in daily environments has yet to be demonstrated.

**Fig. 1.**
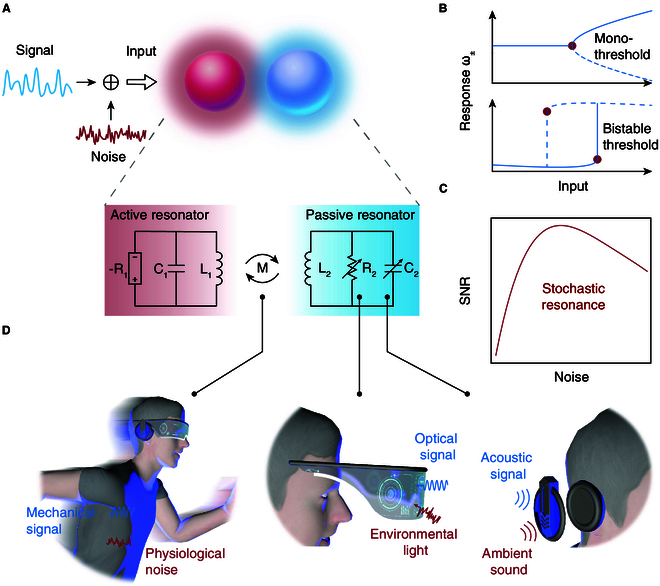
Multi-type stochastic resonances for mechanical, optical, and acoustic sensing. (A) Illustration of general mechanism to design multi-type stochastic resonances (SRs). The input consists of a periodic signal superimposed with environmental noise. The system consists of a pair of coupled inductor–capacitor (LC) resonators, with an amplifier and a resistor connected to each resonator. (B) Coexistence of 2 types of sensory thresholds: mono-threshold and bistable threshold. (C) Conceptual illustration of the signature of SRs: SNR is maximized by a non-zero noise level. (D) Schematic photos for mechanical, optical, and acoustic sensing in daily environment.

Here, we propose a sensor architecture with the simultaneous presence of multi-type SRs and demonstrate their applications in mechanical, optical, and acoustic sensing domains. Our system comprises a pair of wirelessly coupled inductor–capacitor (LC) resonators, each connected to an amplifier or a resistor in parallel, respectively (Fig. 1A). This configuration has yielded a singularity point in the spectrum under the input of coupling strength or resistance [18–20], featuring a nonlinear sensory threshold [21–23]. In addition, by operating the amplifier in the saturation region [24–26], our circuit has shown bistable sensory thresholds in response to capacitance input [27,28] (Fig. 1B). Leveraging these sensory thresholds, we further elucidate the coexistence of excitable SR and bistable SR in our system (Fig. 1C). We demonstrate mechanical, optical, and acoustic sensing using multi-type SRs in noisy environments (Fig. 1D) and achieve noise-enhanced signal-to-noise ratio (SNR) by 9 dB, 3 dB, and 7 dB, respectively.

## Results

We illustrate the coexistence of excitable SR and bistable SR in the wireless system shown in Fig. 1A. The circuit model consists of 2 inductively coupled LC resonators, where one, connected to an amplifier, acts as the active resonator, while the other, connected to a resistor, functions as the passive resonator. Applying Kirchoff’s current law, we have:

IL,1+V1R1+iωC1V1=0IL,2+V2R2+iωC2V2=0, (1)

where *V_n_* are the voltages and *I*_*L*, *n*_ are the currents in the inductors. *L_n_* denotes the inductances, and *R_n_* stands for the resistances (with *n* = 1, 2). A negative resistor −*R*_1_ is realized via a negative impedance converter utilizing an amplifier. The resonant frequencies of 2 resonators, prior to coupling, are given by $\omega_{n}=\frac{1}{\sqrt{L_{n}C_{n}}}$. Considering a mutual inductance *M* between 2 coupled inductors, the voltages and currents are related as follows:

V1V2=iωL1MML2IL,1IL,2. (2)

The balance equation can be obtained by combining Eqs. 1 and 2:

iωC1+1R1−L2iωM2−L1L2MiωM2−L1L2MiωM2−L1L2iωC2+1R2−L1iωM2−L1L2V1V2=0. (3)

We additionally consider the amplifier operating in the saturation region, where the gain is a function of the power inside the resonator and is described by a commonly used saturation model $g_{\mathrm{sat}}=g_{0}/\left(1+V_{1}^{2}\right)$, with *g*_0_ representing unsaturated gain. The resonant frequency of the system can be directly determined by setting the determinant of the impedance matrix of Eq. 3 and solving for the complex frequencies *ω*. Theoretical characterization of such a system and Monte Carlo simulations of excitable SR and bistable SR are detailed in the Supplementary Note and depicted in Figs. S1 to S4.

**Fig. 2.**
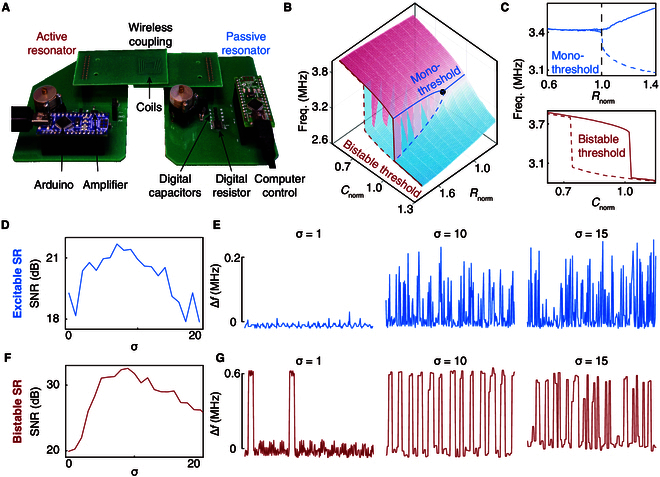
System characterization. (A) A photograph of the proposed wireless circuit system. (B) A full map of resonant frequency in parameter space of *R*_2_ and *C*_2_. The values of *R*_2_ and *C*_2_ are normalized to the singularity point (black dot). (C) Two types of sensory threshold. The singularity point serves as a mono-threshold and the bistability induced by the nonlinearly saturable amplifier acts as the bistable threshold. (D and F) Noise-maximized SNR in excitable SR (D) and bistable SR (F). (E and G) Representative output waveforms for excitable SR (E) and bistable SR (G) under the algorithm noise levels of *σ*= 1, 10, and 15.

We conduct experimental characterization of the system by measuring the resonant frequency under different values of *R*_2_ and *C*_2_. A photograph of the proposed circuit system is presented in Fig. 2A, accompanied by a detailed circuit diagram illustrated in Fig. S5. The resonators are designed in Advanced Design System (Keysight Technologies). *R*_2_ can be digitally programmed with a range of 75 to 1,000 Ω and 4 Ω resolution (AD5254, Analog Devices). *C*_2_ can be digitally programmed with a range of 12.5 to 194 pF and 0.355 pF resolution (NCD2400M, IXYS Corporation). An operational amplifier (ADA4817, Analog Device), powered by a 3.3-V supply (E3646A, Keysight), is used to construct a negative impedance converter. Arduino boards (Bluno, DFROBOT) and a laptop are used to control *R*_2_ and *C*_2_ using LabVIEW interface (National Instruments). Notably, the system is self-oscillating, and its response can be directly captured by a signal analyzer (N9000B, Keysight) that measures the oscillation frequency of the circuit with the aid of a near-field probe.

We first characterize the 2 types of sensory thresholds in the system. The full map of the system’s resonant frequency under varying values of *C*_2_ and *R*_2_ is depicted in Fig. 2B. Notably, a singularity point (the black dot in Fig. 2B) emerges within the parameter dimension of *R*_2_. Preceding this singularity, the system maintains a constant resonant frequency, exhibiting insensitivity to the input *R*_2_. However, in proximity to the singularity point, a minor perturbation induces a sudden phase transition, thereby rendering the system responsive to the changes in *R*_2_ (Fig. 2C). This singularity thus serves as a mono-threshold: the system evokes an output response only when the input surpasses the threshold, mimicking the behavior in excitable neuron model [3,4]. We mark this type of sensory threshold as “mono-threshold” in our system and term the corresponding SR as “excitable SR”.

We also observe the presence of a “bistable threshold” in the same system, as illustrated in Fig. 2C. This phenomenon arises due to the nonlinearly saturable amplifier [24–28], causing the system’s resonance to exhibit bistability in response to variations in *C*_2_. Within the bistable region, the system may potentially support 2 resonant modes; however, only one resonance stabilizes, contingent upon its prior state. For example, when the system is initially resonant at a frequency in the upper branch, the system prevents the other resonant state in the lower branch from becoming steady until the system leaves the bistable region and switches to the other mode (Fig. S2). Notably, resonant frequency transitions occur solely at the boundaries of the bistable region. This hysteresis effect leads to bistable sensory thresholds and facilitates bistable SR within the system.

We proceed to characterize the simultaneous presence of excitable SR and bistable SR. Either type of SR necessitates 3 fundamental components: a form of threshold, a weak coherent input, and a source of noise. In our experimental setup, we generate weak resistance inputs in sinusoidal form (signal rate, 0.14 Hz) and Gaussian noise (standard deviation *σ*, unitless) of resistance using an algorithm implemented in MATLAB (MathWorks Inc.). Note that other noise types such as uniform noise, Rayleigh noise, and exponential noise are also valid in SRs (Fig. S4). The weak input and noise are then added and programmed into the digital resistor *R*_2_ through a LabVIEW interface (National Instruments) to characterize the excitable SR. A similar algorithm is employed to generate weak sinusoidal capacitance input (signal rate, 0.05 Hz) and Gaussian noise, which are then programmed into the digital *C*_2_ to characterize the bistable SR. The generated signal rates can also be changed by different programming speeds in the LabVIEW interface. The time-varying resonant frequency of the system is continuously monitored and processed by fast Fourier transform to obtain the power spectrum. The SNR is then calculated by 10log_10_(*S*/*N*_0_), where *S* denotes the power spectral density at the signal rate and *N*_0_ is the background noise.

The experimental results showcasing excitable SR and bistable SR are displayed in Fig. 2D and F, respectively, revealing the signature of SRs: the maximal SNR occurs at a non-zero noise level. The optimal noise levels with a standard deviation of *σ* = 7 and *σ* = 9 enhance the SNR of excitable SR and bistable SR by 3.16 dB and 12.68 dB, respectively.

The underlying mechanism can be elucidated through the time-varying response of the system’s resonant frequency. For the system featuring a mono-threshold, the output signal in response to a weak coherent input comprises a sequence of spikes, i.e., the ground state interrupted by short, noise-induced excitation events (Fig. 2E). For the bistable threshold, the system exhibits noise-induced transitions between 2 metastable states (Fig. 2G). When *σ* = 1 (Fig. 2E and G), the noise intensity proves inefficient to drive the system’s resonant frequency to effectively switch between the ground state and the excitation state or between the bistable modes. However, as the noise level increases, the input signal intermittently surpasses the sensory threshold with enhanced coherence (Fig. 2E and G, *σ* = 10). At the optimal noise level, the noise-induced excitation or bistable transition events statistically synchronize with the input signal, as evidenced by the noise-maximized SNR in Fig. 2D and F. Nevertheless, further increasing the noise predominates over the coherence. The output flips too many times to be statistically relevant with the input, thereby diminishing the SNR (Fig. 2E and G, *σ* = 15).

**Fig. 3.**
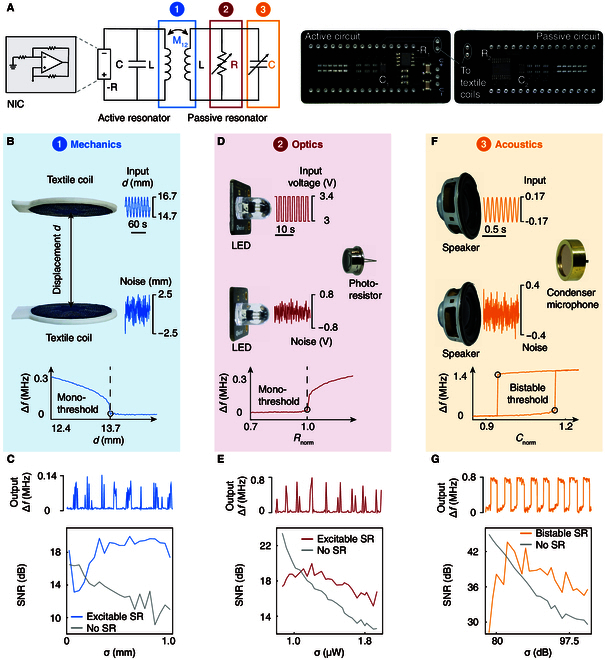
Mechanical, optical, and acoustic sensor demonstrations. (A) The circuit configuration for sensing applications. Mechanical, optical, and acoustic signals are converted to the coupling, resistance, and capacitance signals in the circuit, respectively. NIC, negative impedance converter. (B, D, and F) Experimental setup for mechanical (B), optical (D), and acoustic sensing (F). Inserted curves indicate the sensory thresholds. (C, E, and G) Output signals and SNR as a function of noise in mechanical (C), optical (E), and acoustic sensing (F).

We demonstrate the sensing capabilities of SRs across mechanical, optical, and acoustic signals using the sensors that manifest as inductor, resistor, and capacitor connected to the sensing circuit, respectively. We construct the sensing circuit with fixed resistor and capacitor values, as depicted in Fig. 3A. The inductors are *L*_1_ = *L*_2_ = 5.5 μH, and the resistor and capacitor are set to different values for various demonstrations.

The mechanical sensor measures a coherent mechanical signal through the changes in the coupling strength between the passive and active resonators. The active resonator is attached to a moving stage (KRF4-06-0200A, THK, 0.02 mm resolution) while fixing the position of the passive resonator. An input signal is employed to control the displacement *d* between the 2 coils, and hence the coupling strength, via a motorized linear stage controlled using LabVIEW (National Instruments). The input signals, comprising sinusoids with additive white noise (Fig. 3B), are generated using MATLAB (MathWorks). The circuit yields a mono-threshold occurring at displacement *d* =13.74 mm, beyond which the coupling strength falls below the sensory threshold and the system does not respond.

For comparison, the standard method uses an identical setup with *C*_1_ and negative impedance converter removed. The input signals, the noise, and the control instruments and algorithms are the same as those in the SR sensor. When placed in close proximity to the passive resonator, the reflection spectrum has a dip near the resonant frequency. The response of *L*_1_ is measured by connecting the terminals to a vector network analyzer (N9915A FieldFox, Keysight) using a subminiature version A (SMA) connector and coaxial cable. The output signal is obtained by tracking the dip in the reflection spectrum *S*_11_ induced by proximity to the passive resonator.

Figure 3C shows the response of the system to a sinusoidal input signal of 0.09 Hz superimposed with white Gaussian noise. The sinusoidal signal has an amplitude of 1 mm, which is below the 2-mm gap between the initial position and the displacement threshold. However, the addition of Gaussian noise with a standard deviation *σ* =0.5 mm yields an output featuring a sharp spectral peak at 0.09 Hz, characterized a measured SNR of 18 dB (Fig. 3C). The standard method without SR yields an SNR curve that decreases monotonically with *σ*. The mechanical SR sensor optimally benefits from noise at *σ* = 0.6 mm, surpassing the SNR of the standard method by 9 dB.

We next conduct the optical sensing utilizing excitable SR. The sensor detects a coherent optical signal through the resistance changes of a photoresistor. Our setup in Fig. 3D consists of 2 light-emitting diodes (LEDs) controlled by dc voltages, with one emitting binary white light at 0.2 Hz and the other emitting Gaussian noise. A time-varying dc voltage signal in the form of a square wave and noise are firstly generated by MATLAB code and then programmed to the white LEDs through a dc power supply (E3646A, Keysight) and LabVIEW interface. The input light, combined with noise, is measured by a digital photometer (IF PM, Industrial Fiber Optics) to determine its actual input noise level (Fig. S6). A photoresistor (NSL-6510) is then used to convert the input light signal into resistance changes (Fig. S7), which is connected to the passive resonator in parallel, thereby inducing shifts in the system’s resonant frequency. The output signal, stemming from variations in light intensity, experiences a mono-threshold sensory threshold (Fig. 3D).

For comparative analysis, we implement the standard method employing a commonly used amplifier circuit (Fig. S8). The generated optical signals and noise are the same as those in the SR sensor. The LEDs and photoresistor are in the same positions and settings as the SR sensor. The output voltage signal is measured using a digital multimeter (34461A, Keysight), and the SNR is calculated using the same MATLAB algorithm as the theory.

**Fig. 4.**
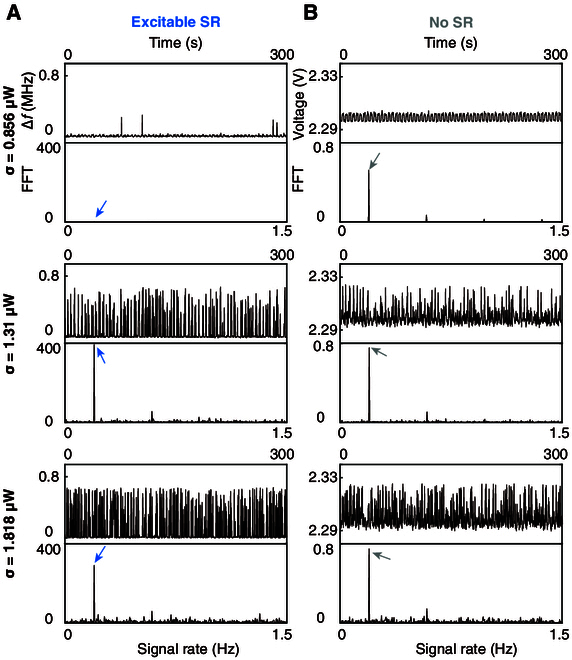
Excitable SR for optical sensing and comparison with standard method. (A) Output signals and fast Fourier transforms of the optical SR sensor under the noise level of 0.856, 1.31, and 1.818 μW. (B) Comparison with standard configuration composed of the commonly used amplifier circuit. The arrows point to the input signal rate. The waveforms of excitable SR indicate the optimized coherence of noise-induced excitation events. However, in the standard method, the noise always deteriorates the input signal and results in low SNR.

The optical sensor demonstrates an output with a maximum SNR of 20 dB at a light noise level of *σ* = 1.3 μW (Fig. 3E). In comparison with the standard method, our optical sensor showcases an SNR improvement up to 3 dB in the presence of high ambient noise. This improvement is attributed to excitable SR, elucidated by the detailed output waveforms depicted in Fig. 4. As the optical noise increases from 0.856 to 1.818 μW, the noise assists the input to surmount the mono-threshold, leading to the random spiking events. Although these spiking events occur at a random time, they exhibit statistical coherence with the input signal at the optimal noise level (second row, Fig. 4A). Conversely, in the standard method, the output transitions from a square-like waveform to a gradually disordered waveform with decreased coherence as noise levels increase (Fig. 4B).

We further demonstrate acoustic sensing using bistable SR. The sensor detects monochrome sounds through the capacitance changes of a condenser microphone. The experimental setup involves 2 input speakers, one generating a 200-Hz sound signal and the other emitting white noise (Fig. 3F). A sound signal at 200 Hz is firstly generated by a MATLAB code and played by one speaker using MATLAB sound function. The Gaussian acoustic noise is generated via random numbers in the MATLAB algorithm and played by the other speaker. The input sound signal is firstly measured by a digital sound meter (FLIR Extech) to characterize the actual sound intensity level (Fig. S9). A condenser microphone and a varactor are then used to convert sound signal and noise into capacitance changes (Fig. S10), which are connected to the passive resonator in parallel. The capacitance changes shift the system's resonant frequency and leads to bistable thresholds (Fig. 3F). We use an oscillator (MDO3012, Tektronix) to record the voltage signal and take a fast Fourier transform to obtain the resonant frequency of the bistable SR system, because the signal analyzer used in mechanical and optical sensor demonstrations is not fast enough to capture the high frequency of the sound. The raw data captured by the oscilloscope at a sound noise of 74 dB, 81 dB, and 89 dB are displayed in Fig. S12.

A standard method is implemented using a commonly used inverting operational amplifier circuit (Fig. S11) and the same condenser microphone. The 200-Hz sound and the noise are the same as those in the SR sensor. The 2 speakers are in the same location as well. The same oscilloscope is used to measure the voltage signal in the standard method.

**Fig. 5.**
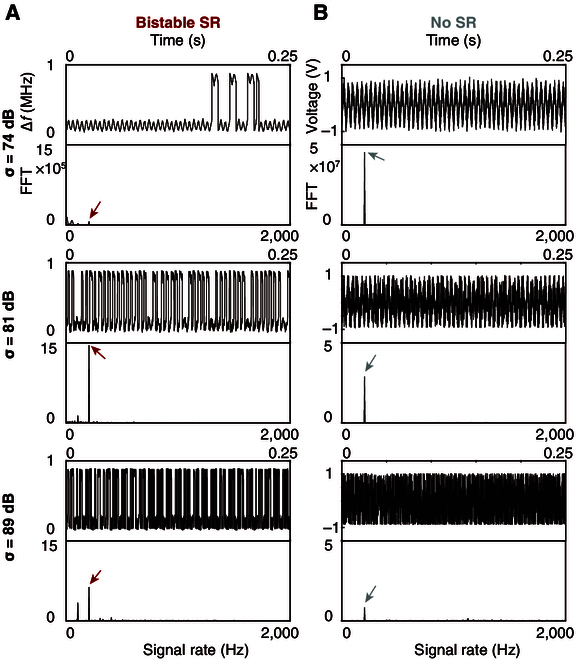
Bistable SR for acoustic sensing and comparison with standard method. (A) Output signals and fast Fourier transforms of the acoustic SR sensor under the sound noise level of 74, 81, and 89 dB. (B) Comparison with standard configuration composed of inverting operational amplifier circuit. The arrows point to the input signal rate. The waveforms of bistable SR indicate the optimized coherence of noise-induced bistable transitions.

The results in Fig. 3G show that, around a sound noise of 81 dB, the circuit output attains a maximal SNR of 42 dB. Comparative analysis reveals that bistable SR leads to a noise-enhanced acoustic detection, characterized by an SNR improvement of 7 dB at the optimal noise level (Fig. 3G). The enhancement stems from noise-assisted bistable mode switching, occurring randomly yet exhibiting statistical coherence with the input sound (Fig. 5A). At a low sound noise, the SNR is high due to frequency shifts within one of the bistable modes. However, as the noise increases (sound noise, 74 dB; Fig. 5A), the system starts hopping between bistable modes, as indicated by the infrequent resonant frequency transitions and a consequent decrease in SNR. At the optimal noise level (sound noise 81 dB, Fig. 5A), the resonant frequency hopping becomes statistically synchronized with the input signal, as evidenced by the noise-maximized SNR, which is the signature of bistable SR. Further escalation of noise (sound noise 89 dB, Fig. 5A) leads to noise dominance over the coherence. The output flips too many times between bistable modes to be statistically relevant with the input. Conversely, in the standard method, the output signal exhibits sinusoidal-like waveform only at low sound noise, with the SNR monotonically decreasing under higher noise conditions (Fig. 5B).

## Discussion

We have introduced a wireless system that supports multi-type SRs and we have demonstrated their widespread applications in mechanical, optical, and acoustic sensing domains. Our approach to multi-type SRs is based on wirelessly coupled electronic resonators, offering versatility for adoption beyond electronic systems, including nanophotonic structures and electromagnetic metamaterials. The proposed method therefore presents a universal mechanism for manipulating signal–noise interactions across diverse sensing systems. Moreover, our experimental demonstrations in mechanical, optical, and acoustic sensing underscore the potential of our multi-type SR system in enhancing the light communication, auditory perception, and physiological monitoring in everyday scenarios. Our method could, in principle, be extended to enhance other coherent signal detections in the Internet of Things, including industrial pressure sensors, environmental monitoring of temperature humidity, and healthcare sensors for electrophysiological recordings, that are enhanced by the noise present in their environments. Our results could also provide some inspirations to other high-related fields such as vibrational resonance and nonlinear resonance [29–31], photonic sensors and signal processors [32–34], and vibration and acoustic signals in electromechanical systems [35–37].

## Materials and Methods

### System design

The sensing circuit is comprised of an active and a passive LC resonator. Textile-integrated inductors weredesigned in software (PE-DESIGN 10, Brother) and fabricated on a cotton-polyester shirt using silver conductivethread (235/34 dtex-super conductive, Shieldex) and computer-controlled embroidery (NV 180, Brother). Theactive resonator used a digitally programmable capacitor (NCD2400M, IXYS Corporation) and a negativeimpedance converter based on a high-speed operational amplifier (ADA4817, Analog Device) with a DC powersupply (E3646A, Keysight). An Arduino Bluetooth board (Bluno, DFROBOT) and a control algorithm implementedin LabVIEW (National Instruments) were used to control the digital capacitor. The resonant frequency of thesystem can be monitored by a signal analyzer (N9000B, Keysight).

### Mechanical sensing

The active resonator was attached to a moving stage (KRF4-06-0200A, THK, 0.02-mm resolution) while fixing theposition of the passive resonator. The moving stage was controlled using LabVIEW (National Instruments). Thestandard method used an identical setup and negative impedance converter removed, interrogated by a vectornetwork analyzer (N9915A FieldFox, Keysight).

### Optical sensing

A white LED was controlled through MATLAB code and a DC power supply (E3646A, Keysight) and LabVIEWinterface. The light noise was measured by a digital photometer (IF PM, Industrial Fiber Optics). A photoresistor(NSL-6510) was then used to perceive the input light. The standard method used a typical amplifier circuit. Theoutput voltage signal was measured by a digital multimeter (34461A, Keysight).

### Acoustic sensing

A sound signal at 200 Hz and noise were played by speakers using MATLAB sound function. The acoustic noiseintensity levels were measured by a digital sound meter (FLIR Extech). The capacitance changes were capturedby an oscilloscope (MDO3012, Tektronix). The standard method was implemented using a commonly usedinverting operational amplifier circuit and measured by the same oscilloscope.

## Data Availability

All study data are included in the article and Supplementary Materials. Pseudocode for the numerical calculation and benchtop experiment is provided in the Supplementary Materials.

## Supplementary Materials

Supplementary 1 Supplementary Note Figs. S1 to S12
