# Supplementary material for: Multi-Type Stochastic Resonances for Noise-Enhanced Mechanical, Optical, and Acoustic Sensing
Source: Research (Wash D C). 2024 May 30;7:0386. doi: 10.34133/research.0386 (PMC11137332; doi:10.34133/research.0386)
Supplement: Supplementary 1 — Supplementary Note Figs. S1 to S12 [file research.0386.f1.pdf]

Supplementary Materials for

Multi-type stochastic resonances for noise-enhanced mechanical, optical, and acoustic sensing

Zhu Liu, Kai Qu, Ke Chen, Zhipeng Li*

*Corresponding author. Email: zhipeng.li@nus.edu.sg

This PDF file includes:

Supplementary Note
Supplementary Figs. S1 to S12

Supplementary Note

Our wireless system can be described by the circuit model shown in Fig. S1a. This circuit can be described by Eq. (3) in the main text. We conduct the numerical analysis by several assumptions to simplify the equation. We first normalize the voltage as $\psi_n = \sqrt{C_n/2}V_n$ such that $|\psi_n|^2$ is the energy stored in the capacitor. Defining the resonant frequencies $\omega_n = 1/\sqrt{L_n C_n}$, loss rate $\gamma_n = 1/R_n C_n$, and coupling coefficient $k = M/\sqrt{L_1 L_2}$, we rewrite Eq. (3) as:

$$\begin{pmatrix} \frac{i\omega}{2}\left[\frac{\omega_1^2}{\omega^2(1-k^2)} - 1\right] - \gamma_1 & -\frac{ik\omega_1\omega_2}{2\omega(1-k^2)} \\ -\frac{ik\omega_1\omega_2}{2\omega(1-k^2)} & \frac{i\omega}{2}\left[\frac{\omega_2^2}{\omega^2(1-k^2)} - 1\right] - \gamma_2 \end{pmatrix} \begin{pmatrix} \psi_1 \\ \psi_2 \end{pmatrix} = 0. \qquad (S1)$$

We make the approximation $k \ll 1$ and $\frac{\omega}{2}\left(\frac{\omega_n^2}{\omega^2} - 1\right) \approx \omega_n - \omega$, choosing to retain the positive frequencies. Considering a gain g by a negative value of γ_1 and time-harmonic voltages $\psi_n(t) \to \psi_n e^{-i\omega t}$, Eq. (S1) reduces to:

$$\begin{pmatrix} i(\omega_1 - \omega) - \gamma_1 & -i\kappa \\ -i\kappa & i(\omega_2 - \omega) - \gamma_2 \end{pmatrix} \begin{pmatrix} \psi_1 \\ \psi_2 \end{pmatrix} = 0, \qquad (S2)$$

where $\kappa = \frac{\omega_1\omega_2}{2\omega}k$ is the coupling rate. We additionally consider the amplifier operating in the saturation region, where the nonlinear gain eliminates the imaginary part of ω. We can therefore plot the numerical solutions of ω with zero imaginary part. The numerical results are shown in Fig. 1b and two types of sensory thresholds, mono-threshold and bistable threshold, are also displayed in Fig. 1c.

Monte Carlo simulations are carried out to characterize excitable stochastic resonance (SR) and bistable SR. We first generate a sinusoidal modulation $m(t) = m \sin \Omega t$, where m is the parameter κ or ω_2 in excitable SR or bistable SR, respectively. We also consider Gaussian noise generated by normal random numbers in the MATLAB algorithm:

$$\xi(t) = N(\mu, \sigma^2) \qquad (S3)$$

where the mean value $\mu = 0$ and σ the standard deviation. Fig. S2a and S2c illustrates the typical output waveforms of excitable SR and bistable SR, which are the noise-induced excitation events and bistable transitions. We calculate the spectrum of the square-like waveform by fast Fourier transform using fft function in the MATLAB algorithm, and we calculate the SNR by $SNR = \log_{10} S(\Omega)/N_0$, where $S(\Omega)$ is the power spectrum at the modulation rate Ω and N_0 is the average background noise. Noise-maximized SNR can be observed in both excitable SR and bistable SR, as shown in Fig. S2b and S2d.

Supplementary Figures

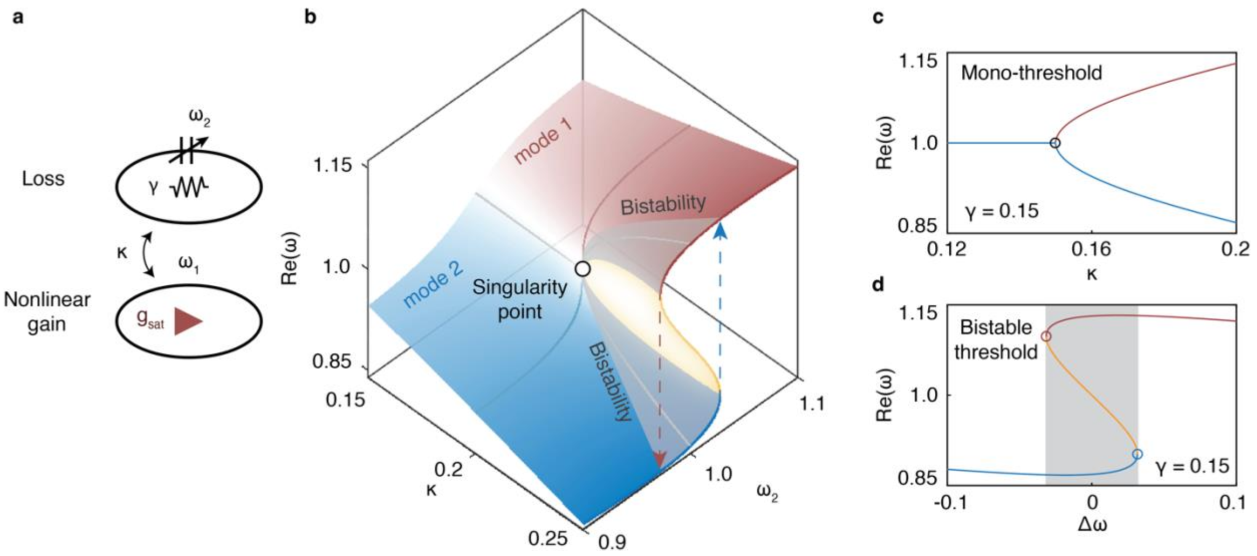

Fig. S1. Numerical analysis of the proposed wireless system. (**a**) Illustration of the proposed wireless system. γ loss rate, g_{sat} saturable amplifier, κ coupling rate, ω_2 detuning. (**b**) The system's resonant frequency under a parameter space of κ and ω_2. (**c-d**) Numerical results of mono-threshold and bistable threshold.

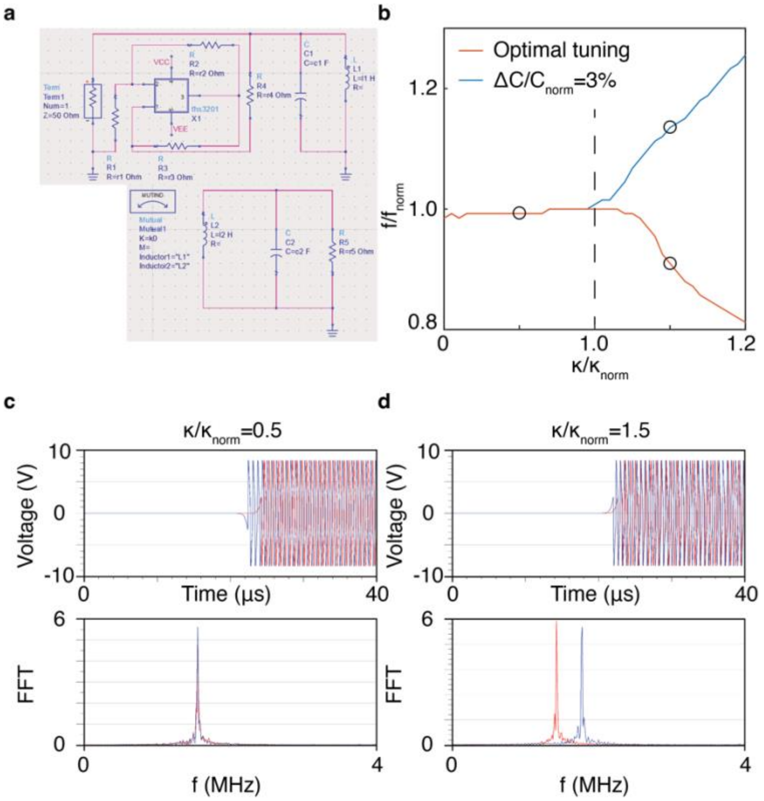

Fig. S2. Circuit simulations of resonant modes with time-harmonic voltages. (**a**) Simulation model in Advanced Design System (ADS, PathWave). (**b**) Resonant frequencies near mono-threshold when the capacitance is in optimal tuning or slightly detuning ($\Delta C/C_{norm}$ = 3%). (**c,d**) The time-harmonic voltages and the spectra before the mono-threshold (κ/κ_{norm} = 0.5) and after the mono-threshold (κ/κ_{norm} = 1.5).

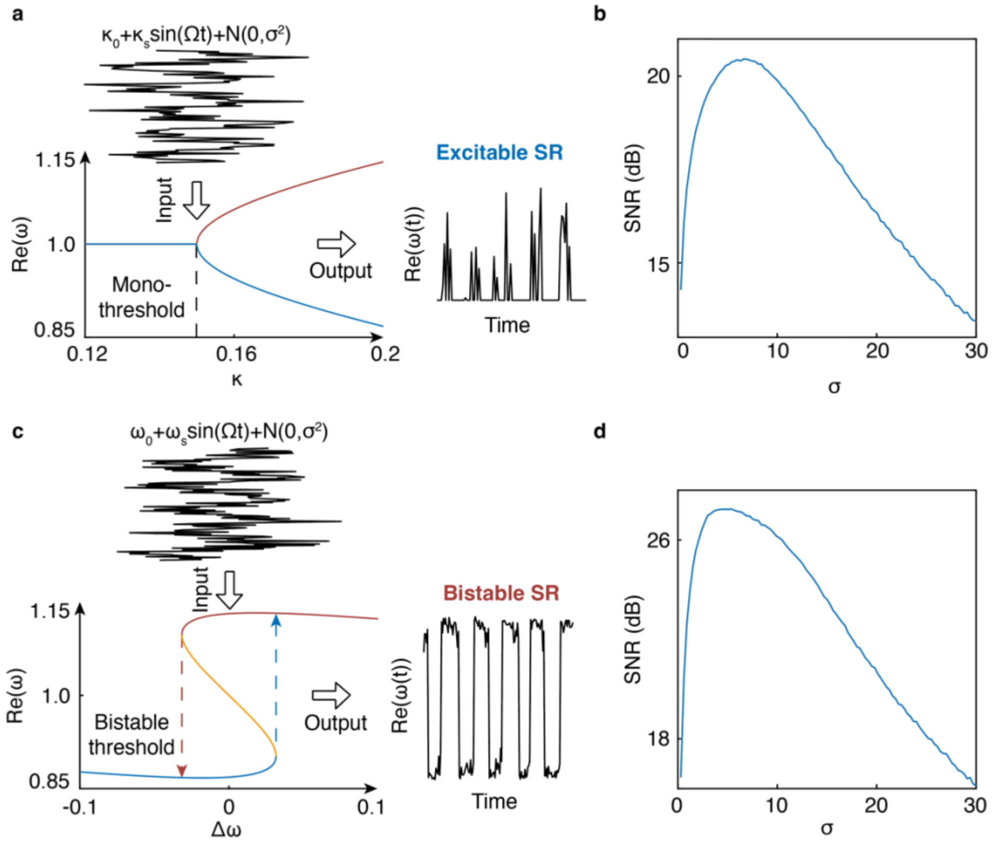

Fig. S3. Simulations of excitable SR and bistable SR. (**a**,**c**) Numerical illustration of typical waveforms of excitable SR (**a**) and bistable SR (**c**). (**b**,**d**) SNR under different σ for excitable SR (**b**) and bistable SR (**d**). Both SRs show noise-maximized SNR.

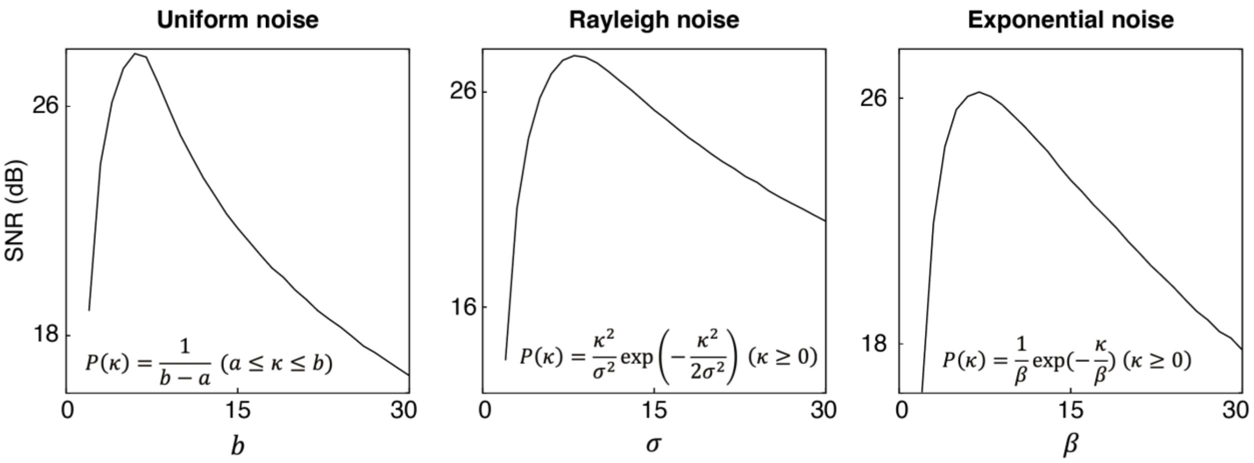

Fig. S4. Excitable SR under different noise types. The different types of noise are described by the probability density function $P(\kappa)$. All the noise types lead to excitable stochastic resonance indicated by the noise-maximized SNR.

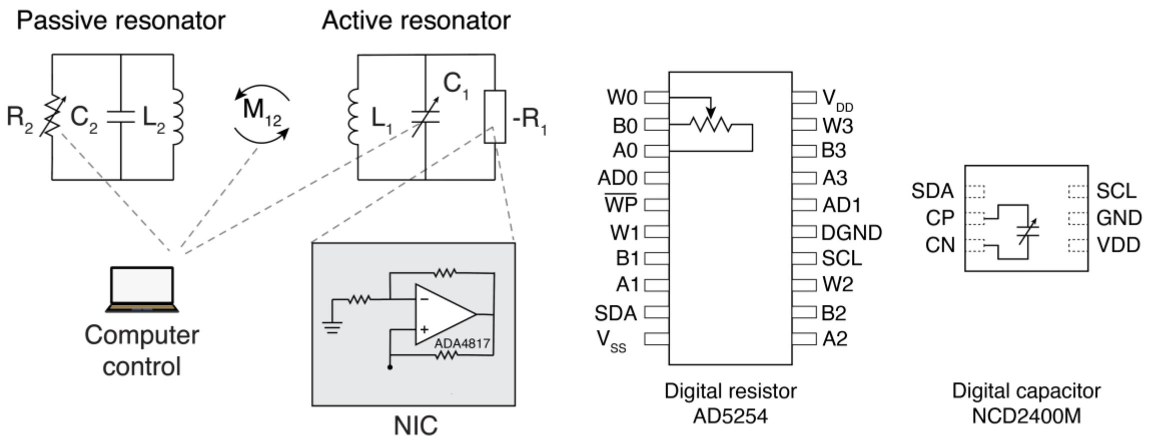

Fig. S5. The detailed circuit diagram. A negative impedance converter is used as $-R_1$. The mutual coupling M_{12}, the resistance in the passive resonator R_2, and the capacitor C_1 are controlled by computer.

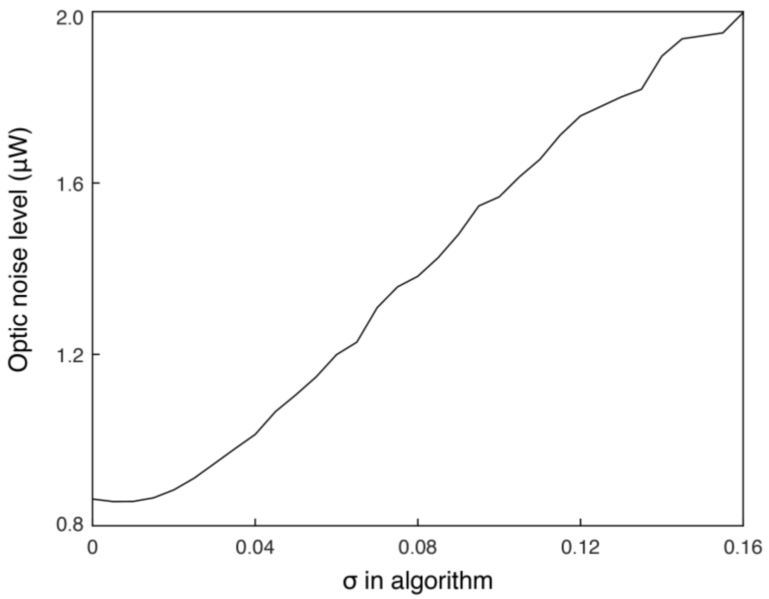

Fig. S6. The actual noise level of white light as a function of standard derivation in algorithm. The results show a linear relation between the actual noise level of white light and the standard derivation of the numerically generated signal.

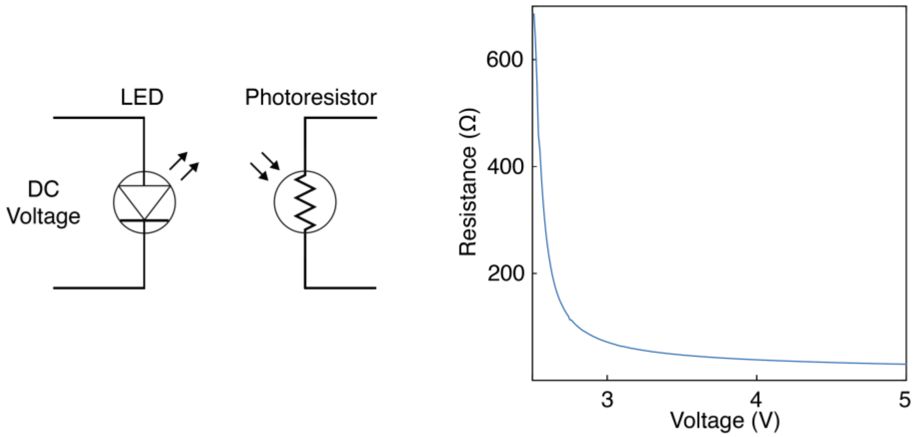

Fig. S7. The resistance of the photoresistor under different voltages of the LED. The input voltages to the LED are controlled to generate white light with different intensity. The resistance of the photoresistor decreases due to the higher light intensity.

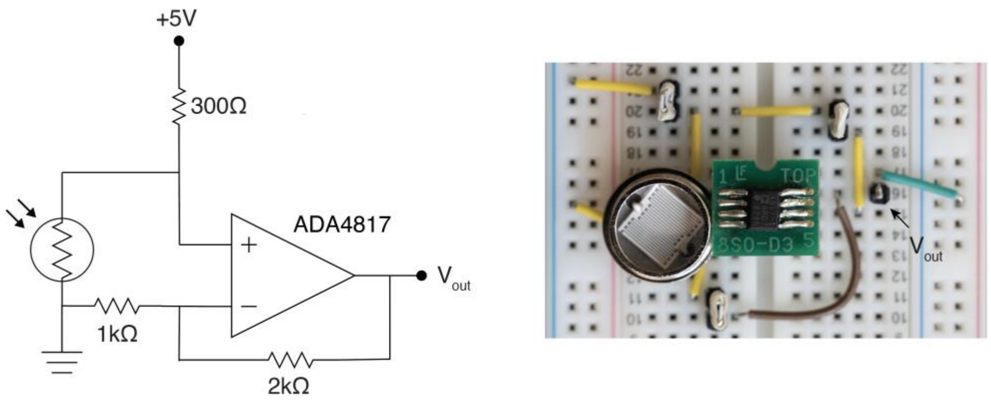

Fig. S8. Optical sensing by the standard method. A standard amplifying circuit for photoresistor measurement is implemented as a standard method, which has no SR. For comparison, the amplifier in this circuit is the same as in the negative impedance converter to ensure that the noise induced by the amplifier are in the same level.

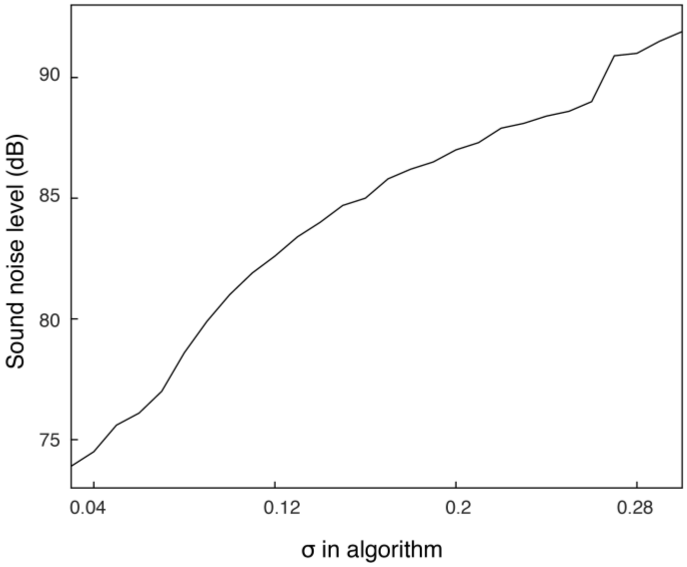

Fig. S9. The actual sound noise level as a function of standard derivation in algorithm. The results show a linear-like relation between the actual noise level of sound signal and the standard derivation of the numerically generated signal.

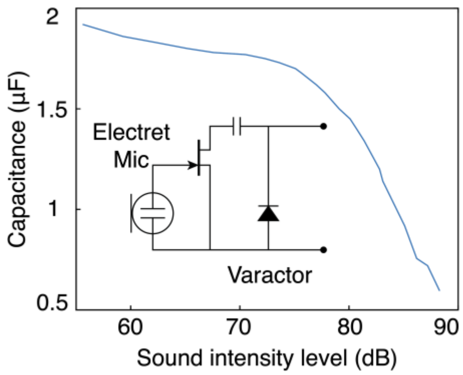

Fig. S10. Converting sound signal into capacitance changes in the circuit. The noisy sound is measured by a condenser microphone, amplified by a built-in amplifier, and converted to capacitance changes using a varactor. The varactor as a tunable capacitor is connected in parallel to the circuit. The results show the average capacitance as a function of sound intensity levels.

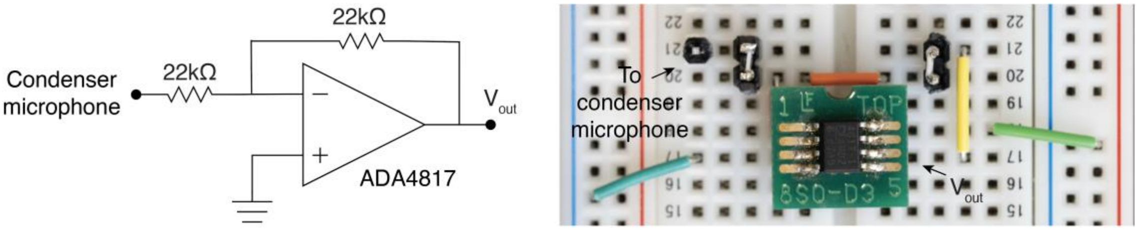

Fig. S11. Standard method for acoustic sensing. The standard method for acoustic sensing is implemented by an inverting operational amplifier circuit that is commonly used in commercial acoustic system, which has no SR. For better comparison, the amplifier in this circuit is the same as in negative impedance converter to ensure that the noise induced by the amplifier are in the same level.

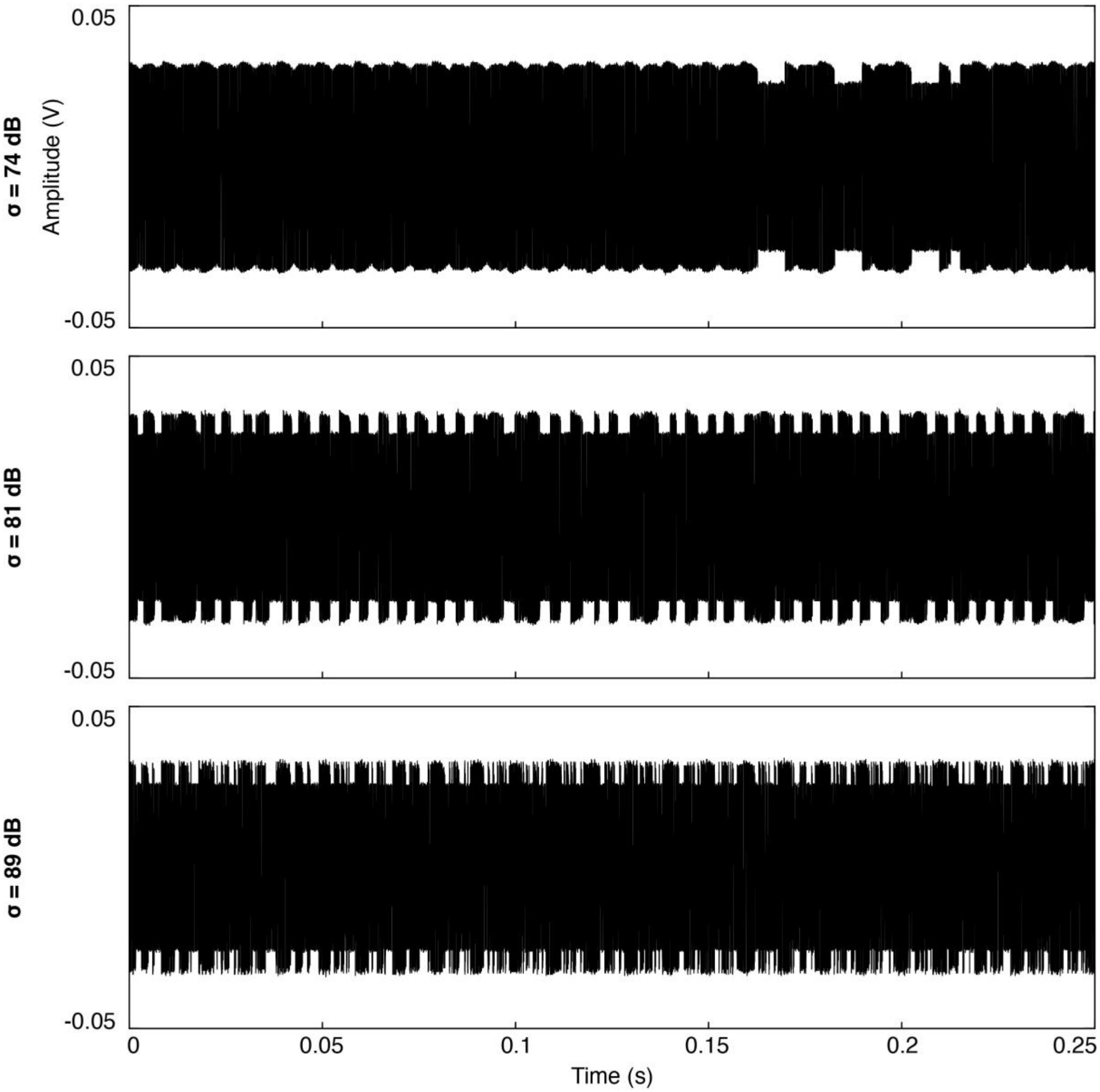

Fig. S12. Voltage amplitudes in the resonant circuit under different sound noise. The voltage amplitudes have a sequence of sudden changes randomly in time due to noise-induced bistable transitions.
